# Concern for overtreatment using the AUA/ASTRO guideline on adjuvant radiotherapy after radical prostatectomy

**DOI:** 10.1186/1471-2490-14-30

**Published:** 2014-04-07

**Authors:** Jung Hun Kang, Yun-Sok Ha, Sung Kim, Jihyeong Yu, Neal Patel, Jaspreet S Parihar, Amirali Hassanzadeh Salmasi, Wun-Jae Kim, Isaac Yi Kim

**Affiliations:** 1 Section of Urologic Oncology, Rutgers Cancer Institute of New Jersey, Rutgers, The State University of New Jersey, 195 Little Albany Street, New Brunswick, NJ 08903, USA; 2 Department of Urology, Samsung Medical Center, Sungkyunkwan University School of Medicine, Seoul, South Korea; 3 Department of Urology, School of Medicine, Kyungpook National University Medical Center, Daegu, South Korea; 4 Department of Radiation Oncology, Rutgers Cancer Institute of New Jersey, Rutgers, The State University of New Jersey, New Brunswick, NJ, USA; 5 Department of Urology, Sanggye Paik Hospital, Inje University College of Medicine, Seoul, South Korea; 6 Department of Urology, Chungbuk National University College of Medicine, Cheongju, South Korea

**Keywords:** Radical prostatectomy, Radiotherapy, Biochemical recurrence

## Abstract

**Background:**

Recently, three prospective randomized trials have shown that adjuvant radiotherapy (ART) after radical prostatectomy for the patients with pT3 and/or positive margins improves biochemical progression-free survival and local recurrence free survival. But, the optimal management of these patients after radical prostatectomy is an issue which has been debated continuously. The object of this study was to determine the necessity of adjuvant radiotherapy (ART) by reviewing the outcomes of observation without ART after radical prostatectomy (RP) in patients with pathologic indications for ART according to the American Urological Association (AUA)/American Society for Radiation Oncology (ASTRO) guideline.

**Methods:**

From a prospectively maintained database, 163 patients were eligible for inclusion in this study. These men had a pathological stage pT2–3 N0 with undetectable PSA level after RP and met one or more of the three following risk factors: capsular perforation, positive surgical margins, or seminal vesicle invasion. We excluded the patients who had received neoadjuvant hormonal therapy or adjuvant treatment, or had less than 24 months of follow-up. To determine the factors that influenced biochemical recurrence-free (BCR), univariate and multivariate Cox proportional hazards analyses were performed.

**Results:**

Among the 163 patients, median follow-up was 50.5 months (24.0-88.2 months). Of those men under observation, 27 patients had BCR and received salvage radiotherapy (SRT). The multivariate Cox analysis showed that BCR was marginally associated with pre-operative serum PSA (*P* = 0.082), and the pathologic GS (HR, 4.063; *P* = 0.001) was an independent predictor of BCR. More importantly, in 87 patients with pre-operative PSA < 6.35 ng/ml and GS ≤ 7, only 3 developed BCR.

**Conclusions:**

Of the 163 patients who qualified for ART based on the current AUA/ASTRO guideline, only 27 (16.6%) developed BCR and received SRT. Therefore, using ART following RP using the current recommendation may be an overtreatment in an overwhelming majority of the patients.

## Background

Prostate cancer (PCa) is the most common noncutaneous cancer in men in the United States [1]. Most newly diagnosed patients present with clinically localized tumor and undergo radical prostatectomy (RP) [2]. However, approximately 20% of patients treated with RP have adverse pathologic features, defined as positive surgical margins (PSMs), extracapsular extension (ECE), seminal vesicle invasion (SVI), and/or lymph node invasion (LNI) [3,4]. Recently, in these patients with high-risk pathologic factors, the American Urological Association (AUA) and American Society for Radiation Oncology (ASTRO) have jointly recommended that adjuvant radiotherapy (ART) be offered [5].

The support from the AUA and ASTRO for ART are largely based on three prospective randomized trials that have shown that ART after RP for the patients with pT3 and/or PSMs improves biochemical recurrence-free survival [6-8]. Although the results of these trials support the benefit of ART for selected patients, a recent treatment patterns analysis showed that ART are applied in less than 20% of the patients with adverse pathologic characteristics and most patients were closely observed with serial PSA tests and offered salvage RT (SRT) only when there was a rise in PSA [9,10]. This reluctance to widely adopt ART might be attributed to the bias of urologic surgeons on the perceived toxicity of radiotherapy. Moreover, recent data demonstrated that patients with PSM who underwent SRT after biochemical recurrence (BCR) had similar long-term outcomes to those who had adjuvant radiotherapy and recurred [11,12].

The optimal role of ART after RP is an issue that has been debated continuously as there is a real risk of complications following radiotherapy [7]. The aim of this study was to evaluate how many men will potentially experience overtreatment using the current ASTRO/AUA guideline for ART. We report that an overwhelming majority of men who qualify for ART do not need the treatment.

## Patients and methods

### Ethics statement

This study was reviewed and approved by the institutional review board of the Rutgers Cancer Institute of New Jersey (RCINJ) (No. 0220080225). Furthermore, the principles of the Helisinki Declaration were followed. The board exempted informed consent because it was a retrospective study.

### Patient selection and clinical follow-up

We reviewed our prospectively maintained database of the 930 patients who underwent RP for clinically localized PCa at the Rutgers Cancer Institute of New Jersey (RCINJ), New Brunswick, NJ by a single surgeon. Using the ASTRO/AUA guideline for ART [5], 163 patients were eligible for inclusion in this study. The inclusion criteria were: pathologic stage pT2–3 N0M0 with undetectable PSA level immediately after the operation and met one or more of the following three risk factors: ECE, PSMs, or SVI. We excluded patients who had received neoadjuvant hormonal therapy or adjuvant treatment, or had less than 24 months of follow-up. All patients were evaluated postoperatively every three months for the first year, every six months for the second year, and yearly thereafter with serum PSA and physical examination. BCR was defined as two consecutive rises in PSA with the last PSA ≥ 0.2 ng/ml.

### Pathologic evaluation

The prostatectomy specimens were processed by having the external surface inked and step sectioned every 4 mm transversely. The prostate apex was examined by sectioning the tissue sagitally. Following staining with hematoxylin and eosin, Gleason score (GS), pathologic stage, and surgical margin status were assessed. Pathologic staging used the 2002 TNM classification. A PSM was defined as the unequivocal presence of tumor at the inked margin of the surgically removed prostate. “Quasi-contact” or “close-by” margins were regarded as negative [13].

### Radiotherapy technique

When required, adjuvant radiation therapy was delivered to the prostate bed using IMRT (intensity modulated radiation therapy) technique, using either a Varian linear accelerator or Tomotherapy machine. The radiation dose and schedule were standard, at 60 Gy delivered in 2 Gy fractions, five days a week. The CTV (clinical target volume) extended inferiorly as low as the superior aspect of the penile bulb; superiorly it extended to just above the pubic symphysis (anteriorly) and incorporated the seminal vesicle remnant (posteriorly). The CTV anterior border was at the posterior pubis symphysis, and the posterior border was the anterior rectum. The PTV (planning target volume), to which radiation dose was actually directed, was made by expanding the CTV by 1.2 cm in all directions, except posteriorly (0.8 cm to spare rectum).

### Statistical analysis

A total of 163 patients were divided into two groups according to the status of BCR and compared in terms of clinical and pathologic data. Independent sample Student’s t-test and the Pearson chi-square test were used to compare continuous and categorical variables, respectively. Kaplan-Meier survival curves were calculated, and the differences were assessed using the log-rank test. The area under the receiver operator characteristic curve (ROC) was used to measure predictive of pre-operative serum PSA levels for BCR yielding the highest combined sensitivity and specificity. Univariate and multivariate Cox proportional hazard models were created to control for predictors of BCR. Hazard ratio (HR) and 95% confidence interval (CI) were computed. Statistical analysis was performed by using SPSS 12.0 software (SPSS Inc., Chicago, IL, USA), and a two-sided *P* value < 0.05 was considered to be statistically significant.

## Results

The median follow-up was 50.5 months (24.0-88.2 months) and the associated demographic data is presented in Table 1. In this cohort, 27 patients had BCR and received SRT during the follow-up period. We divided the patients into two groups according to the status of BCR and compared the clinical and pathologic factors. There were no significant differences in age, prostate volume, PSA density, pathologic stage and PSM rate except for the pre-operative serum PSA level and surgical Gleason score (GS). Compared to the BCR-negative group, BCR-positive group had higher pre-operative serum PSA level (*P* = 0.006). Simultaneously, the incidence of BCR in patients with surgical GS ≥ 8 was significantly higher than that in men with GS ≤ 7 (*P* < 0.001).

**Table 1:** Characteristics of enroll patients

**Variables**	**Biochemical recurrence**		***P***
	**Negative (*N*** **= 136)**	**Positive (*N*** **= 27)**	
Mean (range) age (yr)	60.3 (43-75)	62.6 (52-77)	0.124^a^
Mean (range) PSA (ng/mL)	6.4 (1.4-52.4)	9.0 (2.0-35.6)	0.006^a^
Prostate volume (range) (mL)	43.9 (41.5-46.3)	48.9 (42.0-55.7)	0.172^a^
PSA density (range)	0.16 (0.02-0.87)	0.20 (0.04-0.84)	0.154^a^
Surgical GS (%)			<0.001^b^
6	38 (95.0)	2 (5.0)	
3 + 4	57 (89.1)	9 (10.9)	
4 + 3	18 (90.0)	2 (10.0)	
8	13 (59.1)	7 (40.9)	
9	10 (58.8)	7 (41.2)	
Pathologic stage (%)			0.071^b^
T2a	2 (100)	0 (0)	
T2b	1(100)	0 (0)	
T2c	38 (90.5)	4 (9.5)	
T3a	87 (81.3)	20 (18.7)	
T3b	8 (72.7)	3 (27.3)	
Positive surgical margin			0.459^c^
Negative	66 (85.7)	11 (14.3)	
Positive	70 (81.4)	16 (18.6)	

GS, Gleason score; ^a^Student t-test; ^b^Linear by linear association test; ^c^Chi-square test.

**Figure 1:**
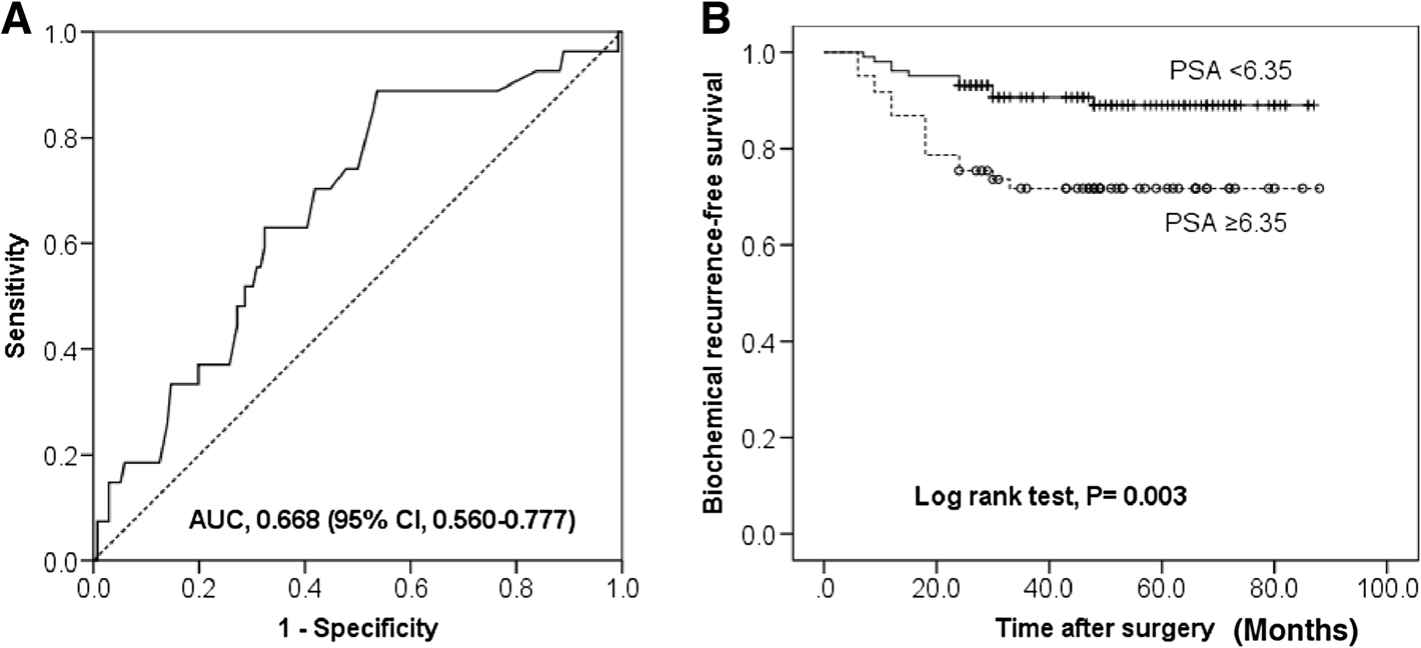
**Pre-operative serum PSA levels correlated with the increased rate of the biochemical recurrence. (A)** Optimal cut-off value of PSA for predicting the biochemical recurrence. **(B)** Biochemical recurrence-free survival according to PSA level.

**Figure 2:**
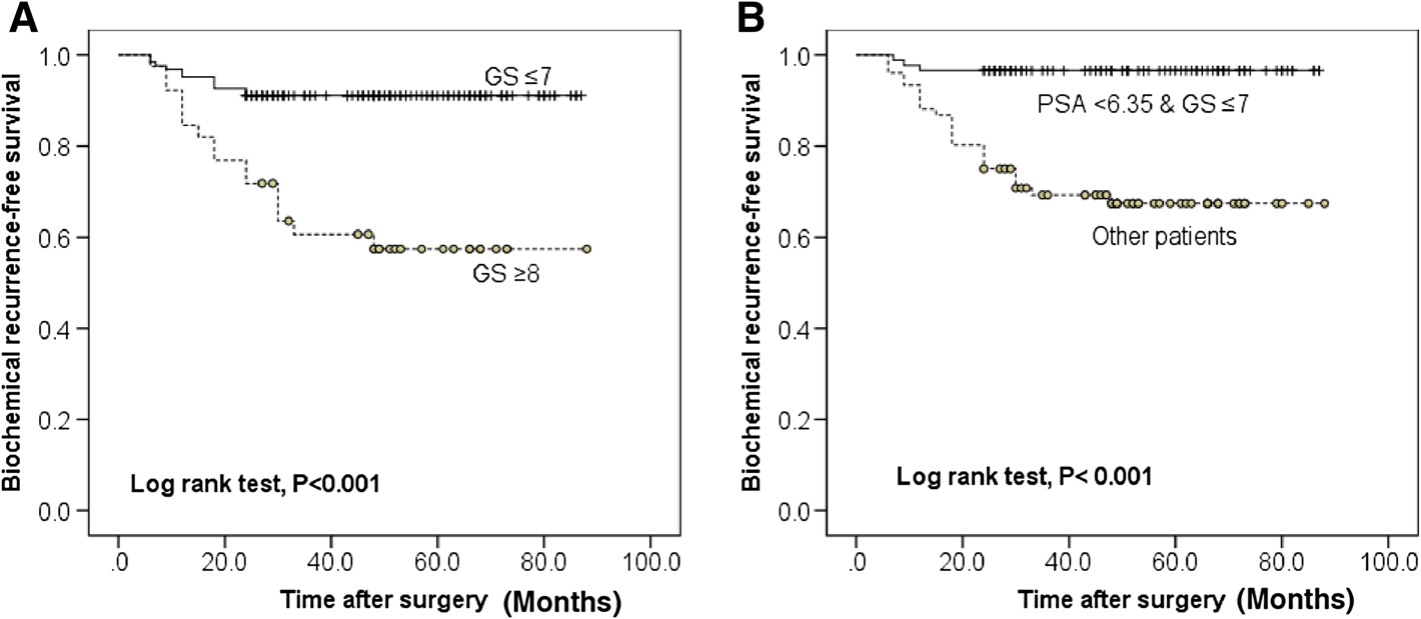
**Kaplan-Meir curves predict the biochemical recurrence. (A)** Biochemical recurrence (BCR)-free survival according to Gleason score. **(B)** Eighty-seven patients who met the criteria composed of lower pre-operative PSA cutoff (6.35 ng/ml) and surgical GS ≤ 7, there were only 3 patients had BCR.

Because pre-operative serum PSA levels correlated with the increased rate of BCR, we examined varying pre-operative PSA cutoffs on BCR. ROC analysis was carried out and AUC of PSA is shown in Figure 1A. PSA level >6.35 ng/ml was shown to be a predictive parameter for BCR (sensitivity 63.0%, specificity 67.6%). Using these values patients were classified into high and low PSA groups. Kaplan-Meier estimates revealed significant differences in time to BCR between the low and high PSA groups (log rank test, *P* = 0.003; Figure 1B). Moreover, patients with GS ≥ 8 were significantly more likely to experience BCR than those with GS ≤ 7 (log rank test, *P* < 0.001; Figure 2A).

By univariate Cox proportional hazards analysis, preoperative serum PSA and surgical GS significantly influenced the time to BCR (Table 2). However, multivariate Cox proportional hazard analysis showed that BCR was marginally associated with pre-operative serum PSA (*P* = 0.082) while the surgical GS (HR, 4.063; *P* = 0.001) was confirmed to be an independent predictor of BCR.

**Table 2:** Cox analyses for BCR

**Variables**	**Univariate**		**Multivariate**	
	**HR (95% CI)**	***P***	**HR (95% CI)**	***P***
Age	1.046 (0.978-1.104)	0.107		
PSA	3.089 (1.414-6.748)	0.005	2.068 (0.911-4.693)	0.082
(<6.35, ≥6.35)				
Post-operative GS (≤7 *vs.* ≥8)	5.077 (2.355-10.944)	< 0.001	4.063 (1.816-9.092)	0.001
Organ confinement	2.235 (0.773-6.463)	0.138		
(T2 *vs.* T3)				
Margin status (Negative *vs.* Positive)	1.419 (0.658-3.062)	0.372		

GS, Gleason score; HR, hazard ratio; CI, confidence interval.

### Patient stratification

Based on the Cox proportional hazard analyses, we re-evaluated the patients according to the risk for BCR. At first we stratified the patients into favorable and unfavorable groups. And the favorable group had GS ≤ 7 and pre-operative PSA 6.35 ng/mL or less. Kaplan-Meir analysis revealed significant differences in the interval to BCR between the favorable and unfavorable group (*P* = 0.001; Figure 2B). More importantly, in 87 patients who met the lower pre-operative PSA cutoff (6.35 ng/ml) and had surgical GS ≤ 7, only three patients recurred.

## Discussion

In this study, we assessed the outcome of observation in men who are recommended to have ART offered following RP based on the current ASTRO/AUA guideline [5]. During 50.5 months of median follow-up period, BCR rate was 16.6%. Therefore, more than 80% of the patients at our institution who qualified for ART based on the guideline did not need ART.

To date, three prospective randomized trials have shown that ART after RP for the patients with pT3 and/or PSMs consistently reduced the risk of BCR anywhere from 50 to 60% and improved the outcome of local control [6-8]. More recently, an update of the SWOG 8794 trial showed improved overall survival with ART when compared to observation [14]. Based on these finding, ASTRO and AUA jointly published a guideline recommending that ART be offered to all men who met the inclusion criteria of the three aforementioned studies. Notwithstanding, the SWOG 8794 trial demonstrated no overall survival benefit for the subset of patients with confirmed undetectable PSA post-operatively [14]. That is, patients who underwent SRT after BCR had similar long-term outcome to those who had ART with undetectable PSA level immediately after RP. Therefore, these results suggest that ART in every man with high-risk features post-operatively is not necessary.

To this end, the present study reported the results of observation without ART in patients who met the ASTRO/AUA criteria for ART. An important finding in our study is that the overall BCR after surgery was very low (16.6%). The prior randomized trials showed a BCR rate of 46% to 61.8% in the observation cohort, respectively. This favorable result may be explained in part by the differences in inclusion criteria and baseline characteristics. Our pre-operative mean serum PSA level was signficantly lower when compared to that of previous studies. For example, the cohort of Wiegel et al. had higher pre-operative PSA level (9.4 ng/ml) than our study patients (6.79 ng/ml) [8]. And the frequency of men with high PSA (>10 ng/ml) was only 14 (8.6%) in our study population. In the trial reported by Thompson et al., the rate of PSA > 10 ng/ml was over 40% [7]. Alternatively, the differences in inclusion criteria may be the underlying reason for the observed low rate of BCR in the present cohort. Specifically, our study analyzed patients who had a pathological stage pT2–3 N0 with undetectable PSA level immediately after RP and met one or more of the three following risk factors: ECE, PMSs, or SVI. Two randomized studies (SWOG and EORTC trials) for patients with pT2 (R1) or pT3 (R0 or R1) disease did not require an undetectable PSA level after RP [6,7]. The third ARO study selected patients with an undetectable PSA level after RP but limited to pT3 tumors [8].

Several studies had shown that GS, initial PSA level, SVI, and PSMs are independent predictors of biochemical progression [15,16]. Our present study also showed that BCR rate was significantly associated with initial PSA level and surgical GS. But, there was no significant association with pathologic stage and surgical margin status. Since the BCR group patients had significantly higher pre-operative PSA level and surgical GS, we performed further analysis to identify factors independently correlated with an increased risk of BCR. The multivariate Cox proportional hazard analysis revealed that GS is an independent predictor of BCR. In the subsequent analysis of the favorable group defined as GS ≤ 7 and pre-operative PSA 6.35 ng/mL or less, BCR was only 3.4%. These results suggest that the criteria for ART based on the ASTRO/AUA guideline need to be re-evaluated to avoid significant overtreatment. We recommend that for patients with pre-operative PSA < 6.35, Gleason score <8, and an undetectable PSA immediately after surgery, observation is a reasonable approach.

Lastly, the present study has a significant economic implication. In the U.S., approximately 90 percent of PCa patients will choose definitive treatment [17], resulting in a projected $12 billion in medical costs in 2010 [18]. At the present time, the most widely used modality for radiation therapy is intensity modulated radiation therapy (IMRT). The calculated reimbursement for IMRT as a primary treatment was $29,356 in patients with low- or intermediate-risk PCa [19]. Other studies reported that the treatment with IMRT costs $15,000–$20,000 more than alternative standard therapies [20,21]. Showalter et al. reported that the mean incremental cost for ART versus observation was $6,023 per patient [22]. Therefore, the minimum direct economic benefit of observation over ART in the present cohort was $819,128 (136 × $6,023).

Potential limitations of the current study are the retrospective study design, relatively small sample size and a relatively short follow–up period. It should be pointed out though, that all data in this study were recorded prospectively. Another limitation is that our study is a single center single surgeon series. Therefore, the impact of surgical technique and institutional bias cannot be assessed.

## Conclusions

The results of our study demonstrated that among 163 patients with a high risk of recurrence based on the ASTRO/AUA guideline, only 27 patients (16.6%) developed BCR and received SRT. In addition, in 87 patients with pre-operative PSA less than 6.35 ng/ml and Gleason score <8, only three recurred (3.4%). Therefore, ART in patients who meet the currently endorsed ASTRO/AUA criteria should be applied more selectively to avoid significant overtreatment.

## Abbreviations

ART: Adjuvant radiotherapy; RP: Radical prostatectomy; AUA: American Urological Association; ASTRO: American Society for Radiation Oncology; BCR: Biochemical recurrence-free; SRT: Salvage radiotherapy; PCa: Prostate cancer; PSMs: Positive surgical margins; ECE: Extracapsular extension; SVI: Seminal vesicle invasion; LNI: Lymph node invasion; GS: Gleason score; ROC: Receiver operator characteristic curve; HR: Hazard ratio; CI: Confidence interval.

## Competing interests

None of the contributing authors have any conflicts of interest including specific financial interests and relationships and affiliation relevant to the subject matter or materials discussed in the manuscript.

## Authors’ contributions

JHK and YSH conceived of the study, carried out the statistical analyses, and draft the manuscript. SK and JY contributed to the interpretation of the results. NP and JSP investigated the clinical records of the considered patients. AHS and WJK contributed to the interpretation of the results and helped to draft the manuscript. IYK designed the study concept, interpreted the results and approved the final manuscript. All authors read and approved the final manuscript.

## Pre-publication history

The pre-publication history for this paper can be accessed here:

http://www.biomedcentral.com/1471-2490/14/30/prepub
